# Szabo 2-stent technique for coronary bifurcation lesions: procedural and short-term outcomes

**DOI:** 10.1186/s12872-020-01605-y

**Published:** 2020-07-07

**Authors:** Hongbo Yang, Juying Qian, Zheyong Huang, Junbo Ge

**Affiliations:** Department of Cardiology, Zhongshan Hospital, Fudan University, Shanghai Institute of Cardiovascular Diseases, Xietu Road No. 1609, Shanghai, 200032 People’s Republic of China

**Keywords:** Coronary bifurcation, Percutaneous coronary intervention, 2-stent technique, Szabo technique

## Abstract

**Background:**

Provisional 1-stent technique is currently regarded as the default approach for the majority of bifurcation lesions. Nonetheless, 2-stent techniques may be required for complex bifurcations with high compromise risk or fatal consequences of side branch (SB) occlusion. Limitations exist in current approaches, as stents gap, multiple metal layers and stent malapposition caused by imprecise placement with fluoroscopic guide and intrinsic technical defects. This study was designed to investigate the effectiveness of the novel Szabo 2-stent technique for coronary bifurcation lesions.

**Methods:**

In the Szabo 2-stent technique, one stent is precisely implanted at the SB ostium with Szabo technique resulting in a single strut protruding into the main vessel (MV). After MV rewiring and SB guidewire withdrawal, another stent is implanted in MV followed by proximal optimization technique, SB rewiring, and final kissing inflation (FKI).

**Results:**

The technique tested successfully in silicone tubes (*n* = 9) with: procedure duration, 31.2 ± 6.8 min; MV and SB rewiring time, 26.8 ± 11.2 s and 33.3 ± 15 s; easy FKI; and 2.3 ± 0.5 balloons/procedure. Bifurcation lesions (*n* = 22) were treated with angiographic success in MV and SB, respectively: increased minimal lumen diameter (0.63 ± 0.32 mm to 3.20 ± 0.35 mm; 0.49 ± 0.37 mm to 2.67 ± 0.25 mm); low residual stenosis (12.4 ± 2.4%; 12.4 ± 2.3%); and intravascular ultrasound confirmed (*n* = 19) full coverage; minimal overlap and malapposition; minimal lumen area (2.4 ± 1.2 mm^2^; 2.1 ± 1.0 mm^2^); plaque burden (78.1 ± 11.3%; 71.6 ± 15.5%); and minimal stent area (9.1 ± 1.6 mm^2^; 6.1 ± 1.3 mm^2^). Periprocedural cardiac troponin increased in 1 asymptomatic patient without electrocardiographic change. There was no target lesion failure (cardiac death, myocardial infarction, target lesion revascularization) at 6-month follow-up.

**Conclusions:**

The Szabo 2-stent technique for bifurcation lesions provided acceptable safety and efficacy at short-term follow-up.

## Introduction

Percutaneous coronary intervention (PCI) of complex bifurcation lesions remains a challenge for interventional cardiologists because of its lower success rate and poor outcomes [1, 2]. Based on studies documenting superiority of provisional 1-stent approach to systemic 2-stent techniques, provisional stenting of the side branch (SB) after main vessel (MV) stenting is currently regarded as the default ‍approach for the majority of bifurcation lesions [3–8]. Nonetheless, fatal consequences can occur from large branch compromise when the provisional 1-stent strategy is unconditionally performed for bifurcation lesions [8, 9]. Therefore, 2-stent techniques should be considered in case of large SB caliber with severe ostial lesion, diffuse lesion, difficult access, or high risk of compromise [9, 10].

A wide variety of 2-stent techniques have been described, including culotte, ‍crush, T stenting and their derivative techniques [1, 2]. Unfortunately, no current technique can avoid the problems of erroneous stent placement, either too far or too proximal with struts protrusion into MV. The ideal technique should provide full coverage with no metal gaps and minimal overlap between the MV and SB stents [11, 12]. Imaging and clinical outcomes have been improved with the development of 2-stent techniques, such as mini-crush, T and protrusion stenting, among others [12–15]. However, technical evolution approached but did not meet the ideal criteria of “full coverage, minimal overlap, and minimal distortion.”

Szabo technique, using a second angioplasty guidewire to anchor the stent at the ostium by passing the proximal end of the anchoring guidewire through the last cell of the stent, has been used successfully in precise positioning of the ostial coronary artery [16–19]. Theoretically, the Szabo technique could be used as a 2-stent technique for bifurcation lesions to facilitate the accurate placement of the SB stent. ‍Here, we describe initial bench testing and clinical application of Szabo 2-stent technique for bifurcation lesions.

## Materials and methods

### Bench testing

The Szabo 2-stent technique was evaluated first in vitro. Tests were performed in silicone tubes (Fig. 4) with different bifurcation angles. Guiding catheter was positioned and guidewires were advanced into both branches.

### Preparation of side branch stent

**Fig. 1:**
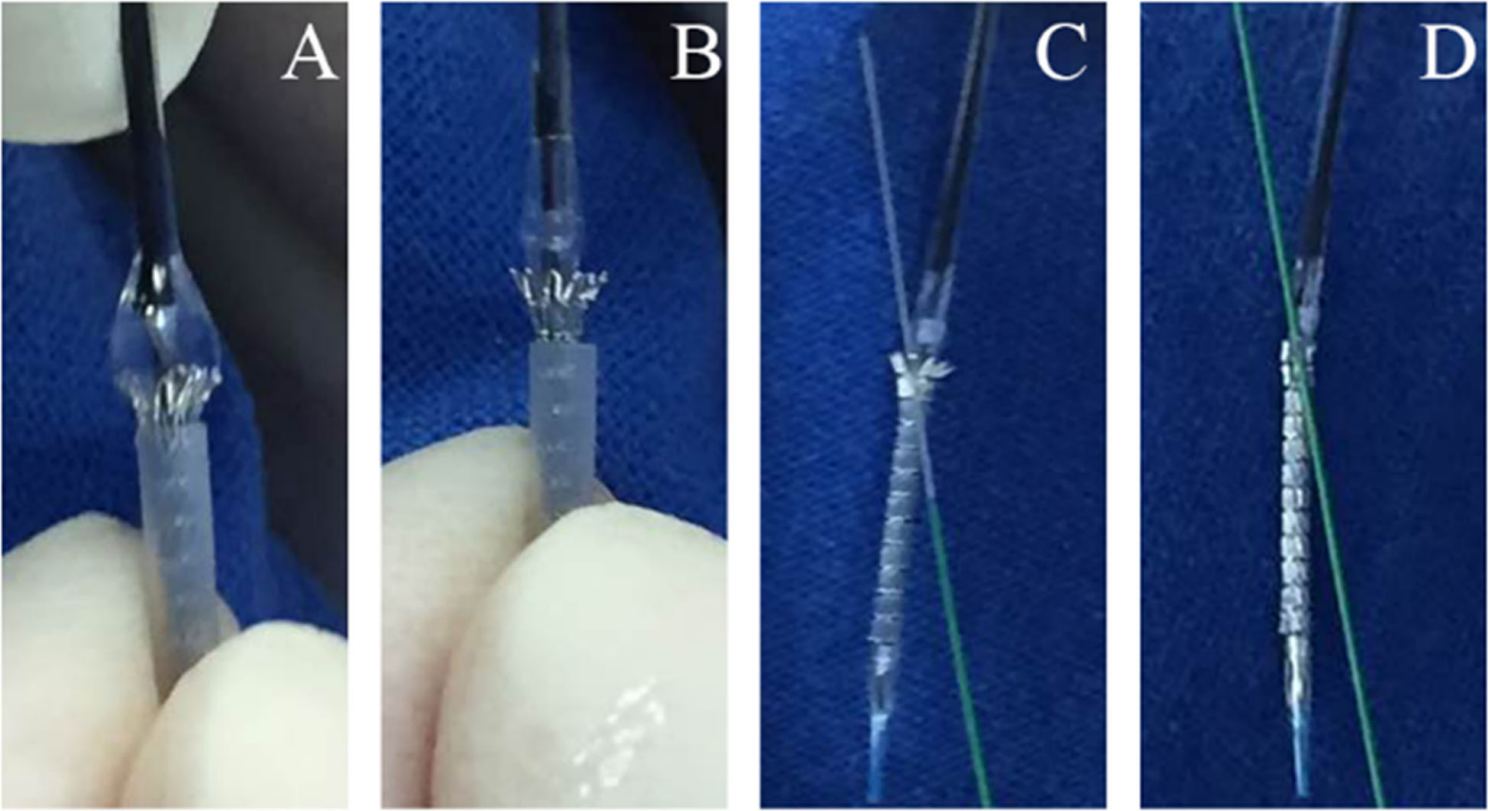
Stent preparation. **a** Inflation with 4 atm. **b** Deflation. **c** Anchoring guidewire threading. **d** Manually crimped flared end of the stent back into place

Preparation of SB stent is shown in Fig. 1. Firstly, the last cell of the stent was lifted up by 4 atm inflation of the stent balloon, with most of the stent remaining inside the protective sheath and only the last cell free (Fig. 1a). When the proximal end of the stent was seen to have flared, the balloon was immediately deflated leaving the last strut free (Fig. 1b). Then, the proximal end of the anchoring guidewire in MV was carefully threaded through the last strut of the stent to avoid balloon injury (Fig. 1c). Finally, the flared end of the stent was manually crimped back into place (Fig. 1d).

### Szabo 2-stent procedure

**Fig. 2:**
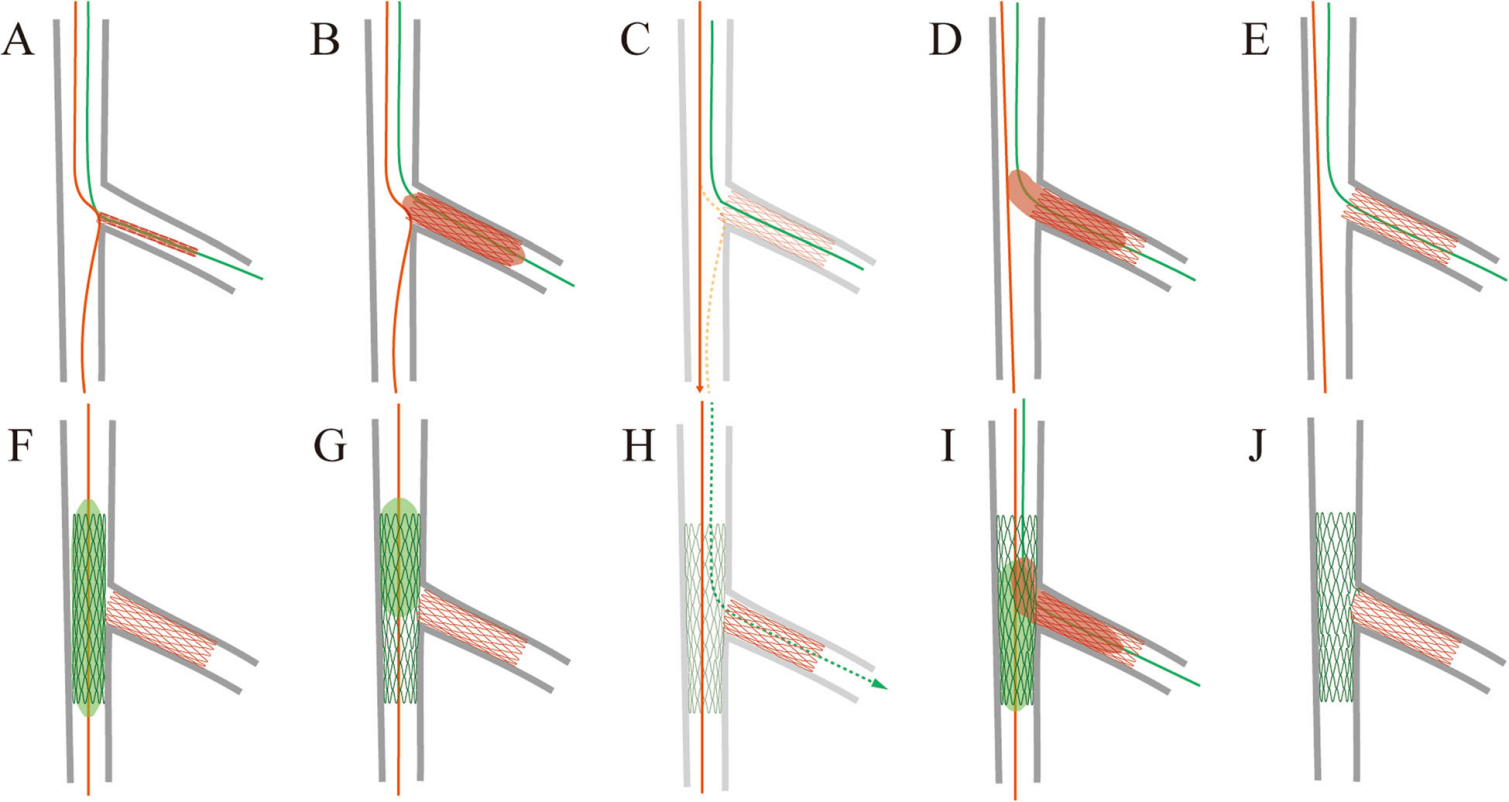
Schematic image depicting the steps of the Szabo 2-stent technique. See detailed explanation in the main text. Guidewires are advanced into the SB (green) and the MV (orange). The SB stent is accurately positioned with anchoring guidewire at the ostial junction (**a**) (see Online Video 1) and deployed (**b**) (see Online Video 2). MV is rewired free from strut (**c**) (see Online Video 3) and SB stent is optimized (**d**). One strut protrudes into the MV (**e**) and the MV stent is positioned and deployed (**f**). MV stent is inflated for proximal optimization technique (**g**). SB rewiring (**h**) (see Online Video 4) and final kissing inflation (**i**) (see Online Video 5) is performed to achieve final results (**j**). MV = main vessel; SB = side branch

With lesion and stent preparation, the SB stent moved freely over both guidewires and was advanced into the SB until further forward movement was blocked by the anchoring guidewire in MV. Coronary angiography confirmed the position of stent at the bifurcation without guidewire wrap. Then final placement was identified by slight bending of the anchoring guidewire at the ostial junction (Fig. 2a) (Online Video 1). At this time, the stent was initially deployed at 8 atm (Fig. 2b) (Online Video 2). The tail guidewire was then easily retracted, and a high-pressure inflation (14 atm) was performed.

**Additional file 1: Online Video 1.** Positioning side branch stent with anchoring guidewire at the ostial junction.

**Additional file 2: Online Video 2.** Inflation of side branch stent.

Next, the guidewire was rewired into distal MV free from strut (Fig. 2c) or another guidewire was advanced into distal MV before SB stent inflation (Online Video 3). SB stent was inflated with stent balloon for optimization (Fig. 2d).

**Additional file 3: Online Video 3.** Advancing another guidewire to distal main vessel before stent inflation is safer than rewiring.

SB stenting with Szabo technique ensured that the stent struts covered lesion completely and was exactly positioned at the ostial SB with only one strut protruding into the MV (Fig. 2e). The MV stent was positioned and deployed (Fig. 2f), followed by proximal optimization technique with a short noncompliant balloon (Fig. 2g).

Then, SB rewiring (Fig. 2h, Online Video 4) and final kissing inflation (FKI) (Fig. 2i, Online Video 5) was performed to obtain best results (Fig. 2j).

**Additional file 4: Online Video 4.** Side branch rewiring.

**Additional file 5: Online Video 5.** Final kissing inflation.

### Patient population

Between November 2018 and May 2019, patients with angina pectoris, attributing to bifurcation lesions without any significant stenosis in other coronary arteries, were selected to undergo treatment using the Szabo 2-stent technique. The bifurcation lesions had > 50% diameter stenosis in both the MV and the ostial SB, and SB stenosis extending for > 5 mm. Of them, severe consequences can occur from the large branch compromise with difficult access. The study was approved by the institutional ethics committee and conformed with the principles of the Declaration of Helsinki. Informed consent for coronary angiography and stent implantation was obtained from all the patients.

### Study procedures and medications

With oral dual antiplatelet drug pretreatment, intravenous bolus of heparin (100/kg) was administered to all patients before angioplasty. Angiograms in multiple views were obtained using the transradial approach, and 2-stent strategies were planned.

**Fig. 3:**
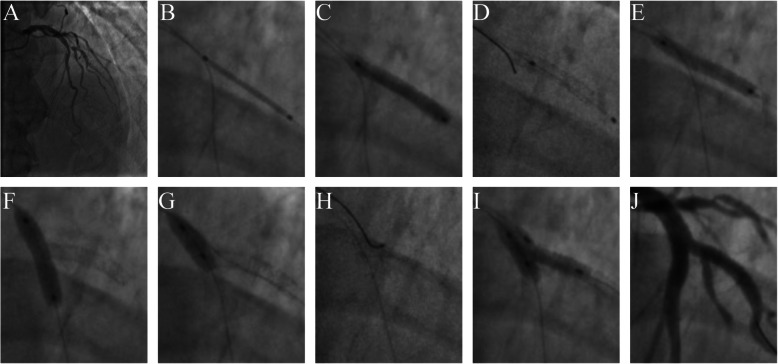
Step-by-step Szabo 2-stent technique application in a patient. Baseline coronary angiogram was presented in (**a**). Diagonal stent is precisely positioned (**b**) (see Online Video 6) and deployed (**c**). LAD rewiring free from strut (**d**) (see Online Video 7) and optimizing diagonal stent (**e**) results in one strut protrusion into LAD (see Online Videos 8 and 9). LAD stenting (**f**) and optimization (**g**) followed by diagonal branch rewiring (**h**) (see Online Video 10) and final kissing inflation (**i**) (see Online Video 11). Final result (**j**) is assessed by coronary angiography (**j**) and intravascular ultrasound (see Online Videos 12 and 13) to confirm the struts gap and overlap. LAD = left anterior descending artery

Guiding catheter was engaged and guidewires were advanced into the diseased arteries. Intravascular ultrasound (IVUS) (OptiCross, Boston Scientific, USA) was performed to confirm the plaque distribution and characteristics. Step-by-step Szabo 2-stent procedure in a patient is illustrated in Fig. 3.

Lesions assessment and preparation were at operator’s discretion. Baseline angiogram was presented in Fig. 3a. With preparation of lesions and stent, the SB stent was accurately positioned (Fig. 3b) (Online Video 6) and deployed (Fig. 3c) at the ostial junction with Szabo technique.

With MV rewiring free from strut (Fig. 3d) (Online Video 7), SB stent was optimized with stent balloon inflation (Fig. 3e). Coronary angiography and IVUS of both branches (Online Videos 8 and 9) were performed to confirm struts coverage and protrusion.

**Additional file 8: Online Video 8.** Full coverage of the diagonal ostium by intravascular ultrasound from the diagonal artery.

**Additional file 9: Online Video 9.** Only 1 strut protrusion into the left anterior descending artery by intravascular ultrasound from the left anterior descending artery.

Next, MV stenting (Fig. 3f) followed by proximal optimization technique (Fig. 3g), and SB rewiring (Fig. 3h) (Online Video 10). Finally, FKI (Fig. 3i) (Online Video 11) was performed to achieve the best results (Fig. 3j). IVUS of both branches (Online Videos 12 and 13) was performed to confirm struts gap and overlap.

**Additional file 12: Online Video 12.** No struts gap and minimal overlap by intravascular ultrasound from the diagonal artery.

**Additional file 13: Online Video 13.** No geographic miss and minimal overlap by intravascular ultrasound from the left anterior descending artery.

### Quantitative analysis

Quantitative analysis was done by an experienced cardiologist. Quantitative angiography analysis was performed by standard techniques with automated edge-detection algorithms (CASS-5.2, Pie Medical, Maastricht, the Netherlands). IVUS imaging was performed after intracoronary administration of 0.2 mg nitroglycerin, using motorized transducer pullback (0.5 mm/s) and a commercial scanner (OptiCross, Boston Scientific, MA, USA). The IVUS data were stored on DVD, and quantitative IVUS analysis (EchoPlaque 3.0, Indec Systems, MountainView, CA, USA) was performed off-line by a single experienced investigator.

### Follow-up and outcomes

Clinical follow-up was performed by office visit or telephone contact at 1, 3, and 6 months. The endpoint was target lesion failure, the composite of cardiac death, myocardial infarction and target lesion revascularization at 6-month follow-up.

## Results

### Bench testing

**Fig. 4:**
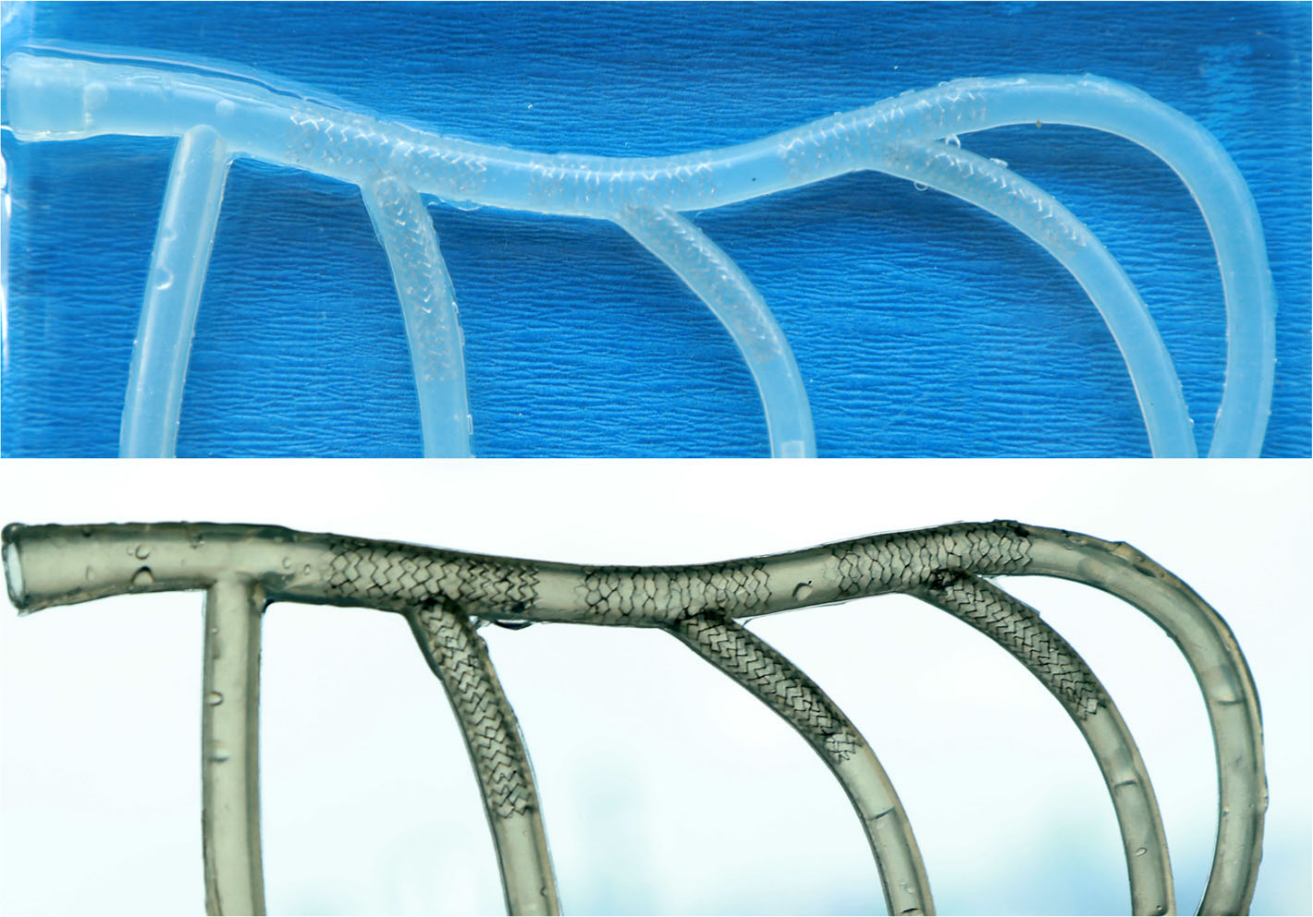
Results of bench testing. Three representative examples from the in vitro procedures show no stent gap with minimal overlap and malapposition of the stents

Bench testing was successfully performed according to the protocol in all 9 procedures with 2 stents per case. Procedure duration was 31.2 ± 6.8 (22–41) min. After SB stenting, MV rewiring time was 26.8 ± 11.2 (15–50) s. Another guidewire was needed to complete the SB rewiring in 1 case, thus average SB rewiring time was 33.3 ± 15.4 (18–70) s. Because of the discrepancy between proximal and distal tube diameters, the initially chosen noncompliant balloons for MV inflation were not suitable for FKI in 3 cases, and on average, 2.3 ± 0.5 balloons were used per procedure. Representative results of 3 cases are shown in Fig. 4.

### Baseline characteristics

Between November 2018 and May 2019, Szabo 2-stent technique was successfully performed in 22 consecutive patients with Medina 1,1,1 bifurcation lesions according to the standardized protocol. The baseline characteristics are summarized in Table 1. Of 22 patients, 16 were male; mean age was 69 ± 11 years; and there was a high proportion of hypertension (81.8%). The indication for PCI was acute coronary syndrome in 10 cases and stable angina in 12 cases. Target bifurcation was located at left main stem in 12 cases and at left anterior descending artery and the diagonal branch in 10 cases. Six cases had mild calcification and none had severe tortuosity.

**Table 1:** Clinical Characteristics of the patients (*n* = 22)

Age, y	69.4 ± 11.1
Male	16 (72.3)
Hypertension	18 (81.8)
Diabetes	2 (9.1)
Hyperlipidemia	2 (9.1)
Smoking	6 (27.3)
Clinical manifestation	
Stable angina	12 (54.5)
Acute coronary syndrome	10 (45.5)
Prior PCI	4 (18.2)
eGFR, mL/min/1.73m^2^	81.2 ± 18.6
LVEF, %	63.1 ± 4.9
Bifurcation	
LAD/LCX	12 (54.5)
LAD/Dg	10 (45.5)
Angle > 70°	10 (45.5)
Calcification	6 (27.3)

Data are presented as mean ± SD or n (%). *Dg* diagonal branch, *eGFR* estimated glomerular filtration rate, *LAD* left anterior descending coronary artery, *LCX* left circumflex coronary artery, *LVEF* left ventricular ejection fraction, *PCI* percutaneous coronary intervention

### Quantitative coronary angiography analysis

Lesion and procedural characteristics are presented in Table 2. Treated branches were large, and diameter of all implanted stents was ≥2.75 mm. Of the 22 stents successfully implanted with Szabo technique, 7 were Promus or Synergy (Boston Scientific, USA), and 5 Xience (Abbott Vascular, USA), 4 Resolute (Medtronic, USA), 3 Firehawk (MicroPort, China) and 3 Excrossal (JW Medical Systems, China). Quantitative coronary angiography analysis showed that mean reference vessel diameters and minimal lumen diameters were 3.29 ± 0.20 mm and 0.63 ± 0.32 mm in the MV, and 2.69 ± 0.31 mm and 0.49 ± 0.37 mm in the SB, respectively. Mean lesion length of the MV and SB was 24.1 ± 12.8 mm and 22.0 ± 16.7 mm, respectively. Mean stent diameter and length were 3.5 ± 0.3 mm and 30.9 ± 16.9 mm in the MV, and 3.0 ± 0.3 mm and 27.3 ± 18.1 mm in the SB, respectively. Maximal inflation pressure was 18.0 ± 4.0 atm and 17.3 ± 3.2 atm in the MV and SB stents, respectively. FKI was performed in all patients with mean inflation pressure of 11.6 ± 0.8 atm. Angiographic success was achieved in all patients with residual stenosis of 12.4 ± 2.4% and 12.4 ± 2.3% in the MV and SB, respectively. Minimal lumen diameters of the MV and SB increased to 3.20 ± 0.35 mm and 2.67 ± 0.25 mm, respectively.

**Table 2:** Quantitative coronary angiographic analysis

	Main vessel (*n* = 22)	Side branch (*n* = 22)
Pre-procedure		
Reference diameter, mm	3.29 ± 0.20	2.69 ± 0.31
Minimal lumen diameter, mm	0.63 ± 0.32	0.49 ± 0.37
Percent diameter stenosis, %	81.8 ± 11.2	82.3 ± 12.4
Lesion length, mm	24.1 ± 12.8	22.0 ± 16.7
Procedure		
Total stent length, mm	30.9 ± 16.9	27.3 ± 18.1
Maximal stent diameter, mm	3.5 ± 0.3	3.0 ± 0.3
Number of stents	1.3 ± 0.5	1.4 ± 0.7
Maximal balloon diameter, mm	3.6 ± 0.4	3.0 ± 0.3
Maximal inflation pressure, atm	18.0 ± 4.0	17.3 ± 3.2
Final kissing inflation pressure, atm	11.6 ± 0.8	
Post-procedure		
Minimal lumen diameter, mm	3.20 ± 0.35	2.67 ± 0.25
Percent diameter stenosis, %	12.4 ± 2.4	12.4 ± 2.3
Acute gain, mm	2.57 ± 0.45	2.18 ± 0.44

Data are presented as mean ± SD. *atm* atmosphere

### IVUS analysis

Three patients refused IVUS examination for financial reasons, so that IVUS was performed in other 19 patients to confirm full coverage, minimal strut protrusion and overlap. Representative IVUS images are shown in Online videos. After diagonal branch stenting, full coverage of ostial diagonal branch and 1 strut protrusion into left anterior descending artery were assessed from both branches (Online Videos 8 and 9). With Szabo 2-stent technique procedure, no struts gap, minimal overlap and malapposition was checked (Online Videos 12 and 13). Quantitative IVUS data are shown in Table 3. Minimal lumen area and plaque burden were 2.4 ± 1.2 mm^2^ and 78.1 ± 11.3% in the MV, and 2.1 ± 1.0 mm^2^ and 71.6 ± 15.5% in the SB, respectively. With successful procedure, minimal stent areas of the MV and SB reached up to 9.1 ± 1.6 mm^2^ and 6.1 ± 1.3 mm^2^.

**Table 3:** Quantitative intravascular ultrasound analysis

	Main vessel (*n* = 19)	Side branch (*n* = 19)
Pre-procedure		
Reference vessel area, mm^2^	10.6 ± 1.8	7.1 ± 1.3
Minimal lumen area, mm^2^	2.4 ± 1.2	2.1 ± 1.0
Plaque burden, %	78.1 ± 11.3	71.6 ± 15.5
Post-procedure		
Minimal stent area, mm^2^	9.1 ± 1.6	6.1 ± 1.3
Acute gain, mm^2^	6.7 ± 1.8	4.0 ± 1.7

Data are presented as mean ± SD

### Follow-up

**Fig. 5:**
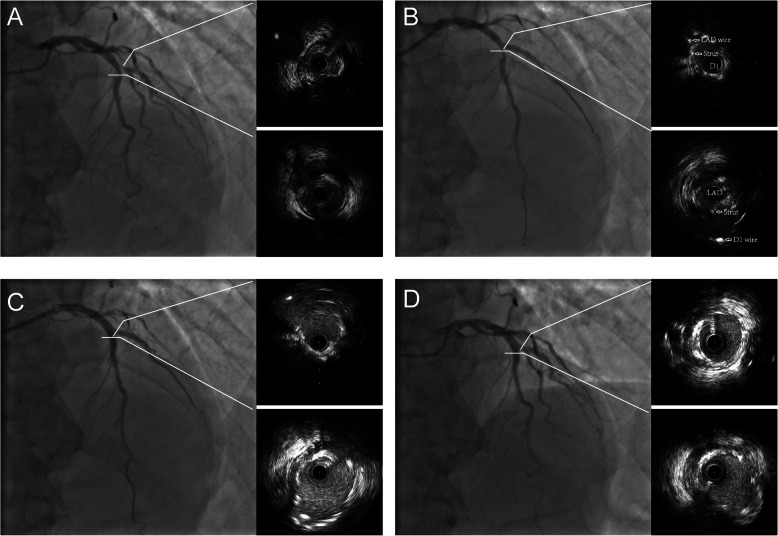
Representative follow-up results of 1 patient. **a** Baseline images with true bifurcation lesion. **b** Accurate placement of stent in the first diagnol branch with 1 strut protruding into the left anterior descending artery. **c** Postprocedure with minimal overlap and malapposition. **d** Follow-up results with mild neointimal proliferation

Cardiac troponin level increased over 5 times the upper limit of the 99% confidence interval in only one asymptomatic patient with no electrocardiographic changes. All patients underwent at least 6-month follow-up (range, 6–11 months). During this period, no target lesion failure was noted, including cardiac death, myocardial infarction, or target lesion revascularization. At a routine visit 11 months after the index procedure, coronary angiography and IVUS in one representative patient revealed only mild neointimal proliferation (Fig. 5).

## Discussion

To the best of our knowledge, this is the first study on use of the novel Szabo 2-stent technique for treatment of bifurcation lesions, which appeared feasible with high rate of procedural success and safe up to 6-moth follow-up.

Coronary bifurcation lesions are frequent and account for approximately 15–20% of total PCIs. Nonetheless, PCI for bifurcation lesions is challenging due to its lower procedural success and poor outcomes. Provisional stenting of the SB is the preferred strategy according to the KISSS principle (Keep it simple, swift and safe) [1–9]. However, the need for bailout stenting in provisional 1-stent approach increases the risks of delivery failure, misplacement or under-expansion of the SB stent, and edge dissection, which could increase the rate of occurrence of significant adverse clinical outcomes driven by target vessel failure and stent thrombosis [6, 20]. Therefore, 2-stent techniques with SB stenting should be considered first in complex bifurcation with difficult access, dissections, high risk of occlusion or need for multiple stents to cover downstream lesions of SB.

Several 2-stent techniques have been developed for bifurcation lesions. T stenting technique was first used with easily applicable and reproducible steps. To decrease restenosis at the ostium of SB stent caused by incomplete coverage of T stenting technique [21], crush and culotte stenting techniques were developed [22, 23]. New problems then arose, such as overlap, multiple metal layers, stent malapposition and under-expansion of stent strut at the ostial SB, which increased restenosis and stent thrombosis [24]. Mini-crush, mini-culotte, double kissing-crush, and nano-crush techniques evolved to minimize overlap and metal burden and to improve clinical outcomes [1, 2, 13–15]. Nonetheless, a significant risk of geographic miss or excessive overlap is inevitably associated with these techniques, which are guided by fluoroscopic placement of the SB stent.

The Szabo technique was first used to accurately position stents in areas with physical barriers; appropriate mechanical placement is important to avoid excessive forward progression at the ostial lesions, reduce the incidence of angiographic malapposition, and ensure full coverage of ostial lesion and minimal protrusion of struts [16–19]. The Szabo technique was associated with favorable outcomes at 2 years follow-up [25]. Thus, the Szabo technique may facilitate precise placement of SB stents, the Szabo 2-stent technique could be used in bifurcation lesions. Here, we investigated the feasibility and safety of the Szabo 2-stent technique for the treatment of bifurcation lesions. During the procedure, after preparation of lesions and stent, the first stent is implanted exactly at the proximal rim of the SB ostium with Szabo technique, resulting in a single strut protruding into the MV. Bending of the anchoring MV wire may happen in some different scenarios like ectatic distal MV or proximal guidewire wrap. Coronary angiography confirms the SB direction and the position of stent at the bifurcation without coronary ectasia or guidewire wrap. Then final placement is identified by slight bending of the anchoring guidewire at the ostial junction to insure exact location. Variety of stent types have been applied in SB ostium with Szabo technique. With MV rewiring and guidewire withdrawal from the SB, the MV stent is positioned and deployed. After proximal optimization technique and SB rewiring at more distal side holes, the procedure finishes with FKI to guarantee potential strut gap in sharp acute angles.

Szabo 2-stent technique might be theoretically superior to many, if not all, 2-stent techniques. First, misplacement of fluoroscopic guide is overcome with anchoring guidewire. Next, it may be superior to crush and culotte stenting with minimal stent overlap of only 1 strut, and better than T stenting with complete coverage of SB ostium. Moreover, Szabo 2-stent technique diminishes malapposition and neocarina formation in T and Protrusion stenting and kissing stenting. Furthermore, easier rewiring and balloon insertion through stent struts could result in higher FKI rate in Szabo 2-stent technique, probably because of the need to cross 1 layer of stent strut versus 3 layers in the crush technique. The novel technique overcomes the vessel diameter discrepancy between the SB and MV in culotte stenting. In addition, different kinds of stents could be successfully used with this technique, which avoids the limitation of single string technique in which cell diameter of SB stent must expand up to at least 4 mm for MV stenting. What’s more, it could be used in provisional 2-stent technique for accurate placement of SB stent as reverse Szabo 2-stent technique. The Szabo 2-stent technique causes minimal deformation of stents (minimal strut protrusion, overlap, malapposition) and vessel structures (full coverage, optimal openings at the ostium, bifurcation angulation), rendering it theoretically ideal to accomodate to the native anatomic structure and preserve its physiological characteristics. Although some of the abovementioned technical and physiological advantages remain to be investigated, it can be anticipated that near-intact anatomy and geometry will translate into pathophysiological and clinical benefits.

In fact, the high procedural success rate and good short-term outcomes demonstrated the feasibility and safety of the Szabo 2-stent technique in this study. A multicenter prospective randomized study comparing the Szabo 2-stent technique with other techniques is warranted to confirm and extend into long-term follow-up the promising results obtained in this single center study.

### Study limitations

The Szabo technique is challenging for some operators. Attention must be paid to complications, such as guidewire entanglement, stent dislodgement and resistance during stent advancement [26, 27]. The operator needs some experience and training to be familiar with this technique, especially be alert to the potential risk of stent displacement from the balloon during stent positioning as stent/balloon integrity may be damaged by the low-pressure inflation. One concern is the risk of comprised blood flow in main branch after SB stenting. A safer method is to advance a new guidewire to the distal MV before withdrawing the anchoring guidewire. If unsuccessful, a small balloon needs to be advanced along the anchoring guidewire to inflate the protruding stent cell, and then the MV stent is deployed across this protruding stent cell, similar to the single string technique [12]. Lack of 3D optical coherence tomography to assess the metallic neocarina and the position of guidewire re-crossing through the MV stent into the SB is another limitation. We tested Szabo 2-stent technique in left main or proximal LAD, but not in more distal bifurcations or in more tortuous anatomies or in smaller anatomies with potential higher risk of wrapping up of the 2 wires. 2-stent strategy is rarely used in more distal bifurcations or in smaller anatomies. Avoiding excessive rotation of wires could help perform Szabo 2-stent technique in tortuous lesions, but the risk of stent dislodgement should be kept in mind. This study could not determine whether the Szabo 2-stent technique is better or worse than provisional or other stenting techniques because of its single center design with short-term follow-up, small sample size, and no control group.

## Conclusions

PCI for bifurcation lesions remains challenging with optimal techniques requiring a balance between complete coverage, minimal stent overlap, and malapposition. In this study, treatment of bifurcation lesions with Szabo 2-stent technique appeared feasible with acceptable procedural success rate, short-term outcomes, and full coverage and minimal overlap and malapposition by IVUS. A large, randomized controlled study with long-term follow-up is warranted to confirm the findings in this study.

## Supplementary information

**Additional file 6: Online Video 6.** Positioning the diagonal stent with guidewire in the left anterior descending artery.

**Additional file 7: Online Video 7.**. Rewiring the left anterior descending artery.

**Additional file 10: Online Video 10.**. Rewiring in the diagonal artery.

**Additional file 11: Online Video 11.** Final kissing inflation.

## Data Availability

The datasets used and/or analyzed during the current study are available from the corresponding author on reasonable request.

## Abbreviations

FKI: Final kissing inflation
IVUS: Intravascular ultrasound
MV: Main vessel
PCI: Percutaneous coronary intervention
SB: Side branch
